# Verrucous carcinoma arising in a long standing Buschke‐Löwenstein tumor

**DOI:** 10.1002/ccr3.2029

**Published:** 2019-02-19

**Authors:** Ahmed G. Elsayed, Saidat T. Sola‐Rufai, Doreen Griswold, Toni Pacioles

**Affiliations:** ^1^ Joan C Edwards School of Medicine, Edwards Comprehensive Cancer Center Marshall University Huntington West Virginia

**Keywords:** Buschke‐Löwenstein tumor, giant condyloma acuminatum, squamous cell cancer, verrucous carcinoma

## Abstract

Giant condyloma acuminatum is a rare variant of genital warts also known as Buschke‐Löwenstein tumor. It is characterized by a slow progression of exophytic, ulcerative, and cauliflower‐shaped tumor with benign histological features. Verrucous carcinoma however is a rare variant of well‐differentiated squamous cell carcinoma with limited metastatic potential.

## 1 CASE

**Figure 1:**
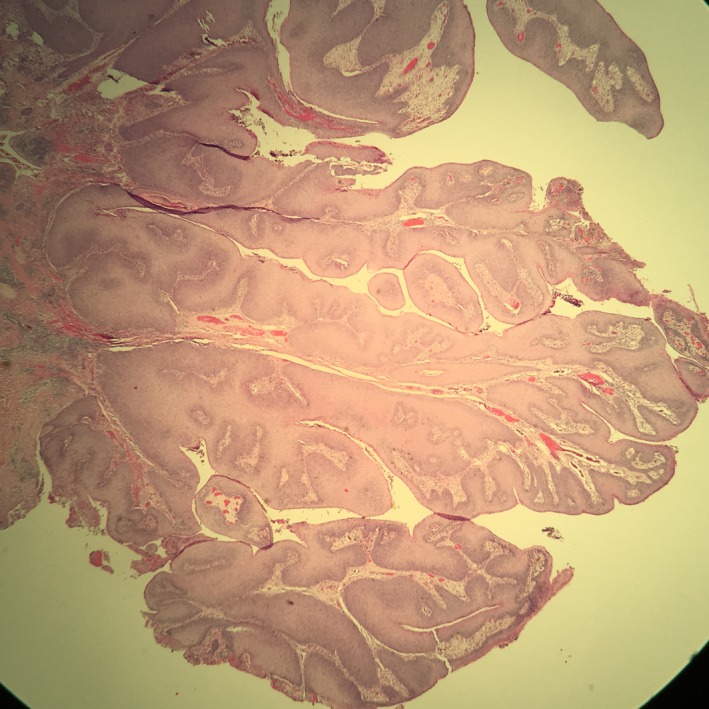
Hematoxylin and eosin stain at 4x power. Cauliflower‐shaped lesion with no invasion to basement membrane consistent with.Buschke‐Löwenstein tumor

A 34‐year‐old African American male with no significant past medical history noticed a lesion in his anal area. This lesion continued to increase in size gradually over seven years and was occasionally tender. He sought medical attention seven years later. At that time, he was diagnosed with anal Condyloma and was referred to surgery. He had a staged surgical resection of his anal condyloma as tumor size was large. Pathologic picture was consistent with giant condyloma acuminata (GCA), also known as Buschke‐Löwenstein tumor (Figure 1).

**Figure 2:**
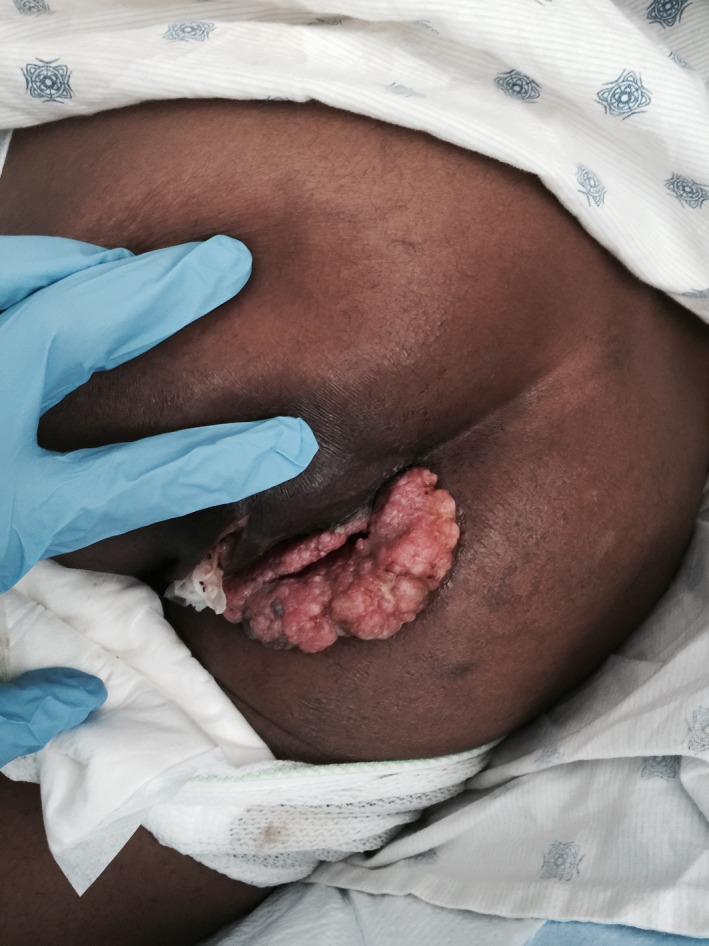
Gross appearance of Verrucous carcinoma status postsurgical drainage of perirectal abscess

**Figure 3:**
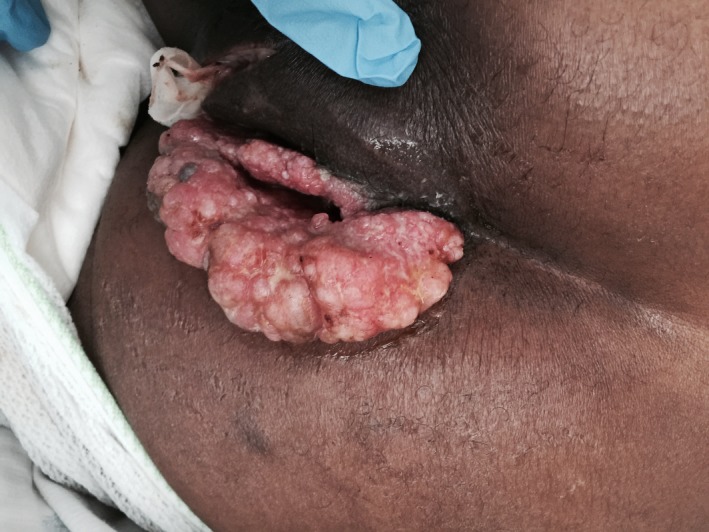
Gross appearance of Verrucous carcinoma

**Figure 4:**
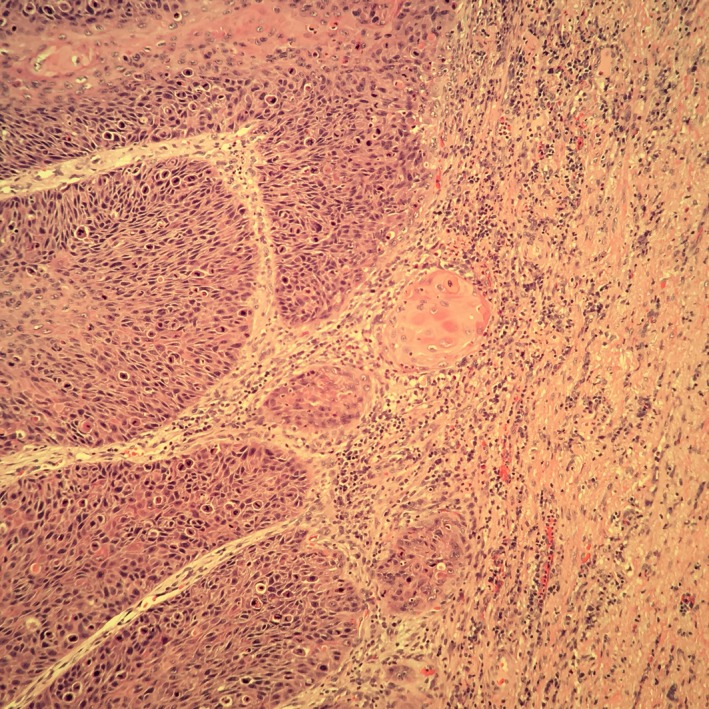
Hematoxylin and eosin stain at 40x power showing well‐differentiated squamous cell carcinoma with underlying chronically inflamed stroma and foci where the basement membrane cannot be clearly visualized, concerning for superficial invasion

Six months after the resection, the lesion grew again in size and patient required further resection. At that time, the lesion had progressed very close to the anal sphincter, and patient was referred to a colorectal surgeon. He was lost to follow‐up for 18 months but eventually presented again with a perirectal abscess and tumor progression (Figure 2 and 3). The abscess was surgically drained. A repeat biopsy done at that time revealed well‐differentiated squamous cell carcinoma with underlying chronically inflamed stroma and foci where the basement membrane is not clearly seen concerning for superficial invasion. The pathological picture was consistent with verrucous carcinoma (Figure 4). The patient was treated with concurrent chemotherapy and radiation. The chemotherapy regimen used was fluorouracil and cisplatin. He was not compliant to treatment. He achieved a partial response and had no progression for 2 years. Upon disease progression, he elected to go for hospice and expired secondary to local progression and subsequent infection.

## 2 DISCUSSION

Giant condyloma acuminatum (GCA) is a rare variant of genital warts. The disease was originally described on a penile foreskin by Buschke and Löwenstein in 1925.^1^ These lesions also occur in anorectal and perianal regions. The GCA also known as Buschke‐Löwenstein tumor is characterized by a slow progression of exophytic, ulcerative, and cauliflower‐shaped tumor. It is also characterized by a lack of spontaneous resolution, local destruction, and direct compression on adjacent tissues and high recurrence rate. The tumors histological features, however, are benign. There is no invasion into the basement membrane.^2^

Verrucous carcinoma is a variant of squamous cell carcinoma that most often arise at anal margin or perianal skin. The tumor usually appears as a well‐differentiated squamous cell carcinoma on microscopic exam. They are typically locally invasive into adjacent chronically inflamed stromal tissue.

## CONFLICT OF INTEREST

None declared.

## AUTHOR CONTRIBUTION

AGE: involved in the conception, acquisition, analysis and interpretation of data, drafted the article or revised it critically for important intellectual content, provided agreement to be accountable for the article and gave final approval of the version to be submitted and the revised version. STS: involved in the conception, acquisition, analysis and interpretation of data, revised the article critically for important intellectual content, provided agreement to be accountable for the article and gave final approval of the version to be submitted and the revised version. DG: collected pathology images, involved in the acquisition, analysis and interpretation of data, provided agreement to be accountable for the article and give final approval of the version to be submitted and the revised version. TP: involved in the conception, acquisition, analysis and interpretation of data, revised the article critically for important intellectual content, provided agreement to be accountable for the article and give final.
